# Advances and Applications of Organ-on-a-Chip and Tissue-on-a-Chip Technology

**DOI:** 10.3390/bioengineering13010009

**Published:** 2025-12-23

**Authors:** Megan Moore, Sashwat Sriram, Jennifer Ku, Yong Li

**Affiliations:** 1 Department of Biomedical Engineering, Western Michigan University Homer Stryker MD School of Medicine, Kalamazoo, MI 49008, USA; megan.moore@wmed.edu (M.M.);; 2 Division of Medical Engineering, Division of Orthopaedic Surgery, Department of Surgical Sciences, Western Michigan University Homer Stryker MD School of Medicine, Kalamazoo, MI 49008, USA

**Keywords:** microfluidics, tissue-on-a-chip, organ-on-a-chip, precision medicine, animal research, translational research

## Abstract

Organ-on-a-chip (OoC) or tissue-on-a-chip (ToC) technologies represent a significant advancement in enabling modeling of human organ and tissue physiology for medical study, although further development is required for these technologies to reach widespread adoption. OoC/ToC are three-dimensional (3D) microfluidic platforms that overcome limitations of traditional two-dimensional (2D) cell culture or animal models, providing an alternative environment for disease study, drug interactions, and tissue regeneration. The design of these systems is complex, requiring advanced fabrication techniques and careful selection of biomaterials with consideration of material toxicity, optical clarity, stability, and flexibility. A key innovation in this field is the multi-organ-on-a-chip (MOC) technology, which links multiple organ systems on a single platform. This enables the study of systemic diseases and the complex communication between organs, which is not possible with single-organ models. Furthermore, OoC/ToC technology holds immense potential for personalized medicine. By using patient-specific cells, these devices can create disease models that reflect an individual’s unique genetic and phenotypic variations, paving the way for tailored therapeutic interventions. The integration of real-time sensors within these devices also facilitates high-throughput screening and accelerates drug discovery. While the development and optimization of these systems is still in its early stages, OoC/ToC technologies have already demonstrated promise in a number of translational research applications.

## 1. Introduction

### 1.1. Overview of the Complicated Nature of Organ-on-a-Chip and Tissue-on-a-Chip Design

Tissue-on-a-chip (ToC) systems are three-dimensional (3D) microfluidic devices engineered to mimic human tissue physiology. Organ-on-a-chip (OoC) systems are similar devices that incorporate multiple cell or tissue types to resemble the higher complexity of organ biomechanics and functions. ToC/OoC technologies mark a critical advancement in research models, allowing for unprecedented precision in replicating human physiologic interactions precisely using in vitro models. These devices provide a revolutionary platform for studying pathophysiology, drug interactions, and tissue regeneration within a controlled and dynamic setting. By utilizing 3D cell cultures, OoC/ToC platforms overcome the traditional limitations of 2D cell-culture technology in both ex vivo and in vivo studies, which often produce unreliable results for translational studies [1,2]. The advantages of these platforms include minimal sample requirements, scalability, predictable fluid dynamics, high resolution and sensitivity, cost effectiveness, high output, and rapid analysis time [2,3,4]. These features make these platforms a compelling alternative to traditional preclinical studies, such as cell culture, significantly accelerating research [5]. The intricate design of these systems, however, poses significant challenges that necessitate a multidisciplinary approach, combining expertise from bioengineering, cell biology, and materials science [6].

The design of OoC/ToC systems involves the recreation of the microarchitecture and microenvironment of human tissues. Human tissues respond variably to different microenvironmental stimuli, often requiring the use of a diverse materials to develop OoC/ToC systems [7]. This process includes the precise arrangement of various cell types, the incorporation of extracellular matrix components, and the integration of mechanical and biochemical stimuli to mimic physiological conditions. Advanced fabrication techniques such as photolithography, 3D printing, and soft lithography are employed to achieve these intricate designs [8,9]. Consequently, the success of an OoC/ToC system is heavily dependent on the choice of biomaterial and fabrication strategies. Historically, silicone has been used in microfluidic systems for its thermal conductivity and flexibility while glass has been used for transparency; however, polydimethylsiloxane (PDMS) has emerged as a widely used material.

PDMS has many advantages with regard to constructing microfluidic systems, but is by no means a perfect material and comes with unique challenges that should be addressed to further optimize microfluidics systems. PDMS is particularly advantageous due to its hyperelasticity, chemical stability, permeability, transparency, and favorable electromagnetic properties [10]. The elasticity of PDMS is useful because it mimics the mechanical properties of biological tissues such as blood vessels and its transparency allows for real-time spectrophotometric observation at wavelengths between 390 nm and 780 nm as well as high-resolution microscope imaging without requiring refractive-index-matching fluid [10,11]. PDMS has also been used in microfluidics to create a linear temperature gradient by applying a heat source and heat sink to opposing sides of a microfluidic device or to create an exquisitely controlled and dynamic temperature gradient across a two-dimensional microfluidics system using sound waves [12].

PDMS systems, despite their advantages, are labor-intensive to construct thus have limited scalability [13]. PDMS additionally can interfere with experiments via either absorbing small hydrophobic molecules placed inside of it or leeching out uncured polymers, thus contaminating the media. The stability of PDMS is also of concern as it may degrade in the presence of some organic solvents or wear down over time and with mechanical stimulation [14]. Maintaining cellular viability and functionality over extended periods requires sophisticated perfusion systems to deliver nutrients and remove waste products, adding further complexities to the engineering process [15].

**Figure 1:**
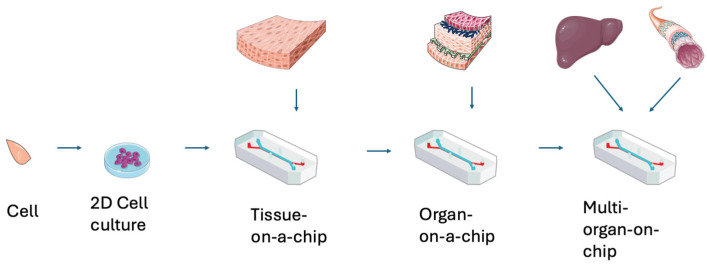
Progression of complexity from cell models to multi-organ-on-chip models. Image(s) provided by Servier Medical Art (https://smart.servier.com), licensed under CC BY 4.0 (https://creativecommons.org/licenses/by/4.0/).

OoC technology can be further developed to more closely resemble human physiology by simulating interactions between different organs, as seen in Figure 1. This requires the functional integration of multiple organ systems on a single chip, termed “multi-organ-on-chip” (MoC). This platform is designed to support cross-organ communication for the study of multiorgan processes, modeling systemic diseases [16]. Consequently, MoC requires the development of interconnecting channels that allow for the communication of biochemical signals and the exchange of metabolites, closely mirroring the interconnected nature of human organ systems [17]. MoC shows promise in replicating human physiological responses in vitro with more nuance even than OoC technologies, but importantly, the complexity, costs, and material requirements are all considerations when constructing MoC systems that may significantly limit their scalability. Studies have demonstrated a system in which human heart, liver, bone and tissue niches are linked by recirculating vascular flow, enabling simulation of interdependent organ functions such as filtration of toxins through the liver [18]. This integrated approach holds the potential to provide holistic and personalized models for understanding and treating multisystem diseases, thereby enhancing the relevance and application of OoC technology.

### 1.2. Significance of Integrating Innovative Technology in Regenerative Medicine

The integration of OoC/ToC technology in regenerative medicine holds transformative potential, promising to overcome several limitations of traditional in vitro and in vivo models [19]. A significant challenge in preclinical studies arises from the inability of animal models to accurately predict therapeutic responses in humans [20,21]. OoC/ToC systems offer a more accurate representation of human physiology, enabling the study of disease mechanisms and drug responses with unprecedented fidelity. This is particularly crucial for diseases with complex pathophysiology, such as neurodegenerative disorders, cardiovascular diseases, and cancer. These miniaturized models have been used to simulate the intricate interactions within human tissues, such as gut-on-a-chip for microbiome and absorption [22], brain-on-a-chip for blood–brain barrier [22], heart-on-a-chip for electrophysiological functions [23], and liver-on-a-chip for metabolism [24].

One of the most significant contributions of OoC/ToC technology is in the realm of personalized medicine. By using patient-derived cells to construct these systems, researchers can create personalized disease models that reflect the genetic and phenotypic variability of individual patients. These “patient-on-a-chip” models are pioneering a new generation of precision medicine. This approach allows for the screening of therapeutic interventions tailored to the specific needs of patients, enhancing the efficacy and safety of treatments [25]. Furthermore, these systems provide a powerful platform for investigating the mechanisms of tissue regeneration and repair. Currently, regenerative therapies for cardiac, respiratory, and hematopoietic are under development with strong potential for translational studies [26]. Despite their numerous benefits, these systems have yet to be used directly as a tool in personalized clinical care.

The ability to integrate sensors and real-time monitoring systems within OoC/ToC devices further enhances their utility. These integrated sensors can continuously monitor key physiological parameters, providing real-time data on cellular responses to various stimuli [27]. Sensors can be used to detect transepithelial resistance for real-time monitoring of barrier integrity; detect pH, oxygen, and lactate to monitor metabolic changes; detect pressure in tissue mechanics monitoring; or detect a number of other biomarkers or changes within systems [28,29]. This capability facilitates high-throughput screening and accelerates the discovery of new therapeutic agents. Additionally, the scalability of OoC/ToC technology makes it a valuable tool for large-scale drug screening and toxicity testing, significantly reducing the reliance on animal models and improving the predictability of human responses [28]. The development of OoC/ToC systems has enabled long-term monitoring of chronic drug responses, as demonstrated by the assessment of dose-dependent toxicity of doxorubicin in dual-organ chip systems [30]. However, performing sensitive in vitro and real-time measurements remains a challenge. Key issues include the small size of OoC/ToC systems and the potential interference of measurement systems with the physiological properties of the biological system under study [31]. This review highlights the translational applications of OoC/ToC technologies, challenges in their implementation, and benefits of OoC/ToC technologies reducing reliance on animal models.

## 2. Applications of These Innovative Technology

### 2.1. Hematology and Oncology Applications

**Figure 2:**
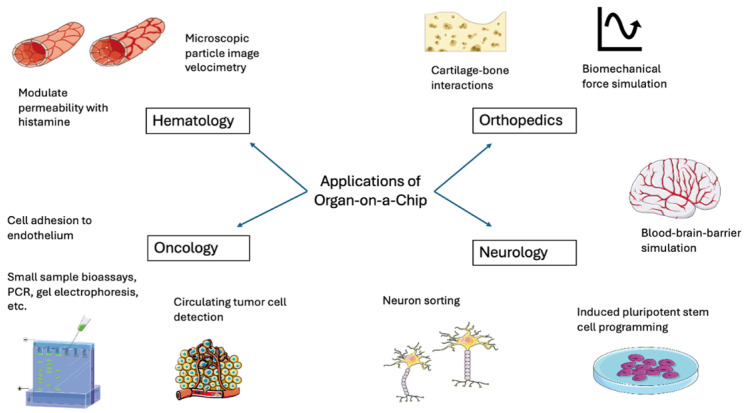
Applications of Organ-on-a-Chip Technologies in Hematology, Oncology, Orthopedics, and Neurology. Applications in hematology include measuring flow velocity and modulating cell–cell permeability to simulate physiologic conditions. Orthopedic applications include studying the interactions between cartilage and bone cell types and simulating biomechanical forces. Oncology applications include several assays and laboratory tests with small samples, simulating cancer cell adhesion to endothelium, and detecting circulating tumor cells. Neurology applications include reprogramming to induced pluripotent stem cells, sorting neuron subtypes, and simulating the blood–brain barrier. Image(s) provided by Servier Medical Art (https://smart.servier.com), licensed under CC BY 4.0 (https://creativecommons.org/licenses/by/4.0/).

Microfluidic systems have enhanced hematology and oncology research by enabling precise simulation of physiologic microenvironment conditions and allowing for biomedical assays using limited sample volume. Figure 2 demonstrates some of these translational applications of microfluidics systems across medical disciplines. In hematology research, microfluidic systems constructed from PDMS have been pivotal in mimicking the dynamic microvascular environment of human blood [11]. Vascular and lymphatic endothelial cells grown in microfluidic devices have been shown to mimic normal physiological functions by forming cell–cell junctions that increase permeability in response to histamine exposure [32]. These systems also enable precise simulation of blood’s non-homogeneous nature consisting of cells and plasma and its variable flow velocities, which are critical for studies on microcirculation and vascular biology [11]. Techniques like microscopic particle image velocimetry (micro-PIV) have been employed within these systems to analyze flow dynamics and cellular interactions in real time, providing insights into hemodynamic forces and their biological impacts [11]. The precise manipulation of cellular interactions and flow dynamics enhances the physiological relevance of microfluidic systems in vascular research, offering clear advantages over traditional static cell culture models. However, additional experimental studies are necessary to further validate these benefits and to refine or modify the systems as recommended.

The field of oncology has also leveraged microfluidic technologies to enhance cancer research, particularly through the development of tumor microenvironment models and tools for non-invasive cancer staging and diagnosis. Microfluidic devices, due to their minute scale and high sensitivity, enable the efficient use of limited sample volumes to conduct a suite of biochemical assays, such as polymerase chain reaction (PCR), gel electrophoresis, microarrays, in situ hybridization, and gene sequencing, thereby economizing the analysis of patient-derived specimens [33]. Microfluidic systems have also been used to isolate circulating tumor cells (CTCs), offering new avenues for understanding and monitoring tumor metastasis. Through immunoaffinity-based isolation, antibodies against the epithelial cell adhesion molecule on cancer cells have been able to tag and isolate CTCs [33,34]. Microfluidics has also been used to isolate CTCs in blood samples by passing blood through microfilters that sort larger CTCs from smaller blood cells. CTC measurement, however, can be difficult due to the rarity of CTCs; CTCs are mostly isolated using epithelial markers, but CTCs can undergo epithelial-to-mesenchymal transition and downregulate these epithelial markers, thus causing poor capture efficiency of CTCs and an underestimation of their prevalence in subsequent measurements [35]. Additionally, CTCs can be heterogenous in marker expression, size, and function, and thus methods that target any of these heterogenous characteristics may miss subsets of CTCs [35]. Microfluidics can monitor cancer cell adhesion to endothelium, which presents an important improvement on traditional research methods that culture cells on static plates [36]. Cells have been shown to express genes and change adhesion properties based on blood flow patterns, so studying cancer cell adhesion in the dynamic environment afforded by microfluidics allows for more physiologically relevant findings [36]. A microfluidic device has been constructed to control flow rates and microscopically monitor in real-time metastatic breast cancer cell adhesion to endothelium in the presence of various chemokines [36].

Limitations and Remaining Challenges in Hematology and Oncology Applications: Microfluidic systems in hematology and oncology face significant limitations, and accurately replicating the heterogeneity of complex blood components with constantly changing flow conditions remains a challenge [11,14]. The material PDMS, while beneficial due to its properties such as elasticity and transparency, is still suboptimal due to its affinity to absorb hydrophobic molecules and leech polymers into experimental media, thus interfering with assay results [14]. In oncology applications, the heterogeneity of circulating tumor cells (CTCs) remains difficult to replicate due to their variable marker expression and inefficient collection and quantification [35]. To address these challenges, future research should focus on developing biomaterials with improved chemical stability to replace PDMS or on finding ways to augment PDMS systems and reduce their interference with experimental media. Efforts should also be made in standardization of hematology and oncology microfluidics systems to improve the scalability and reproducibility of experiments. Lastly, in oncology research, CTC isolation methods that use multi-marker detection to better capture CTCs could improve cell capture and overcome the challenge of low CTC collection.

### 2.2. Orthopedics Applications

Orthopedic research has also benefited from microfluidic technologies, particularly in the study of diverse interactions of three-dimensional bone tissue with other cell types and biomechanical forces subjected to bone that cannot be studied using a traditional static cell plate [37]. Microfluidic technologies, however, allow for the precise manipulation of tissue conditions and organizational structures and thus allow for experimental studies translational to orthopedics [37]. These devices enable precise emulation of the biomechanical forces—such as shear stress, tension, pneumatic compression, and hydrostatic pressure—that bone tissue experiences in vivo. This capability is critical for studying cellular responses like adhesion, proliferation, and differentiation, as well as processes like bone remodeling and tissue regeneration under conditions that closely mimic those in the human body [37]. Microfluidics can be used to detect small amounts of proteins like osteocalcin and metalloproteinase-9 (MMP-9) to help with the cytogenetic characterization and detection of disease. Microfluidic devices can also be constructed to model disease states including intervertebral disc herniation, rheumatoid arthritis, and metastasis of breast cancer to bone [37]. Additionally, microfluidic systems are particularly valuable for modeling the interactions between articular cartilage and subchondral bone, providing insights into arthritis and potential therapeutic approaches. These systems allow for the controlled replication of tissue interfaces and mechanical forces, offering a sophisticated platform for translational studies that aim to bridge the gap between laboratory research and clinical orthopedic applications [38].

Limitations and Remaining Challenges in Orthopedics Applications: Microfluidics systems allow for the precise emulation of some biomechanical forces found in bone and cartilage, but still fall short of replicating the full complexity of in vivo three-dimensional and dynamic forces [38]. The technical complexity required to assemble microfluidics systems for orthopedic research applications as well as cost and labor limit the scalability of these devices for broader research use. To address these challenges, future development should focus on simulating a wider range of biomechanical forces that vary in vector direction and alter over time. Embracing wider use of real-time sensors can also help with dynamic control, better mimicking in vivo conditions that change dynamically.

### 2.3. Neurology Applications

In the field of neurology, the application of microfluidic technologies, often termed “brain-on-a-chip,” represents a significant advancement in the study of neural circuits. Neurons isolated from tissue do not proliferate so engineered neural circuits typically use neural stem cells, which can differentiate into different neuron and glial types [39]. This approach utilizes microfluidics to sort neurons based on hydrodynamic properties, electrophoresis, acoustophoresis, biomarkers, and deformability [39]. Microfluidics improved the feasibility of single-cell RNA sequencing in neural networks and improved cell-type identification, even identifying new subtypes of neurons. Neurons can be classified more accurately and robustly in the microfluidics environment; for instance, neurons can be observed in differing concentrations of calcium and biomarkers for cells based on extracellular environmental conditions [39]. Physiochemical conditions can be harnessed to reprogram neurons into induced pluripotent stem cells (iPSCs). Microfluidics can even determine the polarity of neurons and the growth direction of axons using chemical attractants or repellents to precisely form neuron connectivity patterns [39]. The integration of these neural circuits with multi-electrode arrays (MEAs) provides a robust framework for long-term studies of neuronal activity and three-compartment devices with microchannels for axon and dendrite growth can form synapses to enable studying of neurological diseases caused by synaptic dysfunction [34]. Hippocampal circuits have been engineered using a combination of cells isolated from the entorhinal cortex, dentate gyrus, CA3, and CA1, all connected by microchannels. Additionally, OoC models extend to incorporating elements like the blood–brain barrier, adding another layer of physiological relevance by introducing endothelial cells to mimic blood flow dynamics within the brain [40]. These examples underscore the transformative impact of microfluidic technologies across various biomedical research fields, offering a bridge between in vitro studies and clinical translational research.

Limitations and Remaining Challenges in Neurology Applications: Brain-on-a-chip devices face particularly significant challenges in development due to the complexity and cellular diversity of cell types and interconnected architecture found in the brain [34,39,40]. Inflammatory processes remain suboptimal to study using current microfluidics models as there is a lack of immune system incorporation into current microfluidics platforms. Some of the challenges in these neurology platforms can be addressed with development of new co-culture techniques for diverse neural and glial cells. Development of three-dimensional platforms that support complex tissue architecture and cell–cell interactions is also crucial to advancing neurology microfluidics research. Future research should consider incorporating other organ-on-a-chip systems for multi-organ interactions to better mimic the multifactorial influences found in vivo in neurological diseases.

## 3. Organ-on-a-Chip 3D Structures

### 3.1. Structures

Traditionally, cell culture evaluation of cell behavior involves placing cells in a Petri dish or well plate, producing a 2D cellular organization. However, OoC/ToCs provide a mechanism to evaluate 3D organizations that mimic in vivo conditions more effectively than standard cell culture. These organizations can take on a variety of structures, including spheroids, hydrogels, multilayer, or organoids.

The most simple of these organizations is spheroids, 3D cell aggregates that may be used in ToC systems. Spheroids are first created by clustering cells, allowing for intracellular integrin/cadherin interactions and generation of the ECM [41]. A spheroid consists of three layers including a necrotic core hindered by oxygen/nutrient diffusion, a middle layer consisting of senescent cells, and an outer layer consisting of proliferative cells [42]. Formation of a necrotic core, however, serves limited purposes modeling tissues other than tumor microenvironments. Spheroids may be constructed in the absence of a necrotic core in the modeling of 3D tissue environments with precise control over culture medium, cell properties, and environmental conditions [43].

Hydrogel models, another approach to investigating cellular and tissue behavior, are more physiologically relevant than spheroids when constructing ToC systems as they incorporate an extracellular matrix (ECM) and allow for adjustments in nutrient diffusion and mechanical stimulation [44]. Hydrogels allow for modeling of ECM through facilitation of formation of proteoglycans, collagen, proteins, and integrin binding proteins allowing for generation of ground substance [44]. While spheroids have limitations in nutrient diffusion, hydrogels serve their specialized role in facilitating cell–cell communication and allowing for diffusion of nutrients to central layers of 3D cellular formations in the absence of necrosis.

Cellular multilayer is another approach that allows seeding cells as stacked monolayer scaffolds, generating a 3D structure simulating in vivo conditions [45]. Like hydrogels, multilayers allow for 3D modeling without the presence of areas of necrosis. Multilayers have the benefit of simplicity and replicability. Multilayers have potential in OoC construction as they can model layered tissue such as skin epithelium, respiratory epithelium, and gastrointestinal epithelium [45].

Lastly and most complex are organoids; organoids utilize human induced pluripotent stem cells (hiPSCs), embryonic stem cells (hESCs), and adult stem cells (ASCs) to generate 3D models. Organoids maintain viability while ensuring adequate nutrient and oxygenation of tissue [45]. Organoids may be used with hydrogels to cover the surface of the microfluidic chamber or allow for 3D encapsulation of cellular matrix. Organoids, due to properties of self-renewal, serve as a better choice for investigation of regenerative medicine.

### 3.2. Biomaterials

Using biomaterials such as nanofibrous scaffolds and mats [46] allows for greater control over environmental conditions and cellular organization through greater control of porosity and surface/volume ratio. Natural biomaterials may be generated using gelatin and collagen, while synthetic polymers may be created with compounds such as polyurethane and polystyrene [47]. The combination of multiple synthetic and natural biomaterials allows for greater control over cellular microenvironments.

## 4. Reduction of Animal Testing and Mimicking Mammalian Microenvironments

### 4.1. Microfluidic Systems and Animal Testing

ToC technologies have progressively improved in their ability to replicate in vivo conditions, enabling detailed studies of tissue physiology and disease modeling with a reduced reliance on animal testing. Microfluidic systems simulate the mechanical and biochemical cues of the human body, thereby providing an environment that closely mirrors natural tissue behavior [48,49]. These systems can even be modeled to maintain the physiology of human cells for extended periods, which is essential for long-term studies on disease progression and drug efficacy. A newly developed skin-on-a-chip model, for instance, was able to replicate the epidermis, dermis, and blood vessels in a model that can survive three weeks and could demonstrate the efficacy of dexamethasone in reducing inflammation, thus replacing the need animals in drug screening [50].

Despite these advancements, ToC technologies are still in their infancy and face several challenges compared to traditional animal models. Animal models still generally offer a better representation of the complex interaction between organ systems and require less initial cost than ToC. Animal models have yielded essential data that has guided successful human treatments and studies like the Olson study have provided evidence of the usefulness of animal studies, although this famous study excluded experiments that never entered clinical trials [51].

Additionally, there are some complex processes that even multi-organ-on-chip models cannot fully replace. Systemic immune responses, for instance, require interplay between immune cells with complex cell signaling and trafficking pathways and immune memory that is best replicated in live organisms, although attempts are being made for immune organ study with ToC technologies [52]. Processes that adapt over time such as physiologic pathways in long-term toxicity studies are also challenging to replicate as they feature cumulative effects influenced by whole organism metabolism pathways and repair pathways. Tissue-on-chip technologies must be further advanced before they are able to replicate complex biological systems and must overcome the challenge of limited cell viability in experimental setups [53].

### 4.2. Limitations of Animal Studies

**Figure 3:**
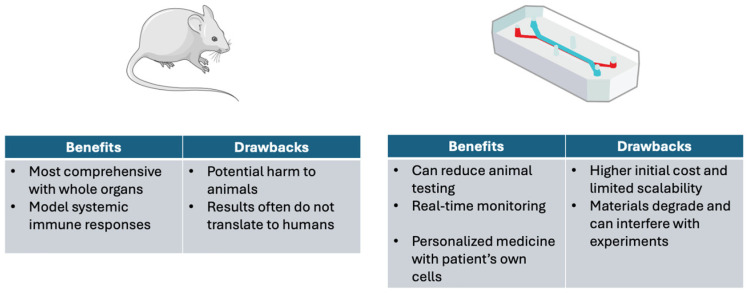
Benefits and drawbacks of animal models versus Organ-on-a-chip technology [14,25,28,29,51,52,53,54,58]. Image(s) provided by Servier Medical Art (https://smart.servier.com), licensed under CC BY 4.0 (https://creativecommons.org/licenses/by/4.0/).

Even animal models, however, are limited in their ability to forecast how in vivo responses will occur when new drugs or altered conditions are introduced into the intricate environment of the human body [54]. A comparison between animal models and organ-on-a-chip technology is provided in Figure 3. Most drugs go through animal testing before being used in clinical trials, yet eight out of nine still prove to be unsuccessful in humans [54]. Drug trials in animals typically assess absorption, distribution, metabolism, and excretion (ADME), but these processes do not always correlate with those in humans due to species differences [55]. Additionally, CRISPR technology, vaccine responses, and monoclonal antibodies that are specifically engineered for humans cannot be effectively modeled in animals. Of historical note, the drug thalidomide proved safe in animal trials but caused severe birth defects in humans [51]. Animal models have sometimes failed to predict the carcinogenicity and toxicity of certain chemicals in humans, leading to preventable cancers in humans. Troglitazone and Rofecoxib were falsely predicted to not be addictive based on animal models [51]. On top of missing negative side effects, relying on animal models can lead to setting aside potential cures as lab animals may not respond to drugs that could benefit humans. Continuing innovations such as multiorgan-on-a-chip and personalized synthetic tissues may lead to ToC being equal or superior to animal testing in an increasing number of laboratory models [56]. Already, considering the development of synthetic systems that can model human physiology better than some animal models, the United States Food and Drug Administration (FDA) Modernization Act 2.0 was passed in December 2022 to remove the requirement for animal testing with certain therapies [57].

### 4.3. Ethical Considerations

Animal testing has also been in popular ethical discourse since 1789 with Jeremy Bentham’s inquiry into animal suffering, which emphasized the importance of the capacity of animals to suffer rather than their cognitive abilities and sanctioned animal experiments under the condition that they served a beneficial purpose to humanity and caused the minimal necessary harm to animals [58]. The predominant approach in animal research ethics today is a focus on minimizing pain, suffering, distress, and lasting harm to experimental animals. The Principles of Humane Experimental Technique emphasize the reduction, refinement, and replacement of animal experiments wherever feasible [58]. With the emergence of ToC technology, more sophisticated in vitro models that better mimic human physiology than animal models are now feasible, thus the ethical framework suggests that research should transition to ToC where possible [54].

## 5. Personalized Medicine and Precision Therapeutics in Cancer Patients

### 5.1. Potential for Personalized Regeneration Techniques

Precision therapy plays a crucial role in the domain of cancer treatment and personalized medicine, as the diverse nature of cancer presents a hurdle in establishing uniform therapeutic strategies. The Somatic Mutation Theory (SMT), originating in 1914 with Boveri et al. and expanded by Bauer et al., posits that cancer results from chromosomal defects or mutations [59,60]. Knudson’s 2-hit theory, an evolution of SMT, suggests two somatic mutations are necessary for carcinogenesis [61]. Multi-Hit Theory, proposed by Ashley et al. in 1969, asserts that 3 to 7 mutations may lead to cancer, with variations in interpretations and notions of hyper-mutation events [62]. The concept of Driver and Passenger Mutations, responding to SMT limitations, distinguishes mutations actively driving cancer (driver) from incidental ones (passenger) [62]. Additionally, acknowledgment of Genetic and Environmental Factors highlights the interplay between mutations and environmental exposures in cancer development [63]. The variability of factors suggesting the development of cancer in vivo proposes the need for a model to establish precision therapeutic approaches to treating cancer on an individual level.

### 5.2. Revolutionizing Precision Medicine Through Patient-Specific Microenvironments

The multifaceted factors contributing to in vivo cancer development underscore the imperative for a comprehensive model to pave the way for precision therapeutic approaches tailored to the individual. OoC systems provide three-dimensional models in vitro that replicate in vivo conditions that may serve the purpose for developing personalized anticancer therapies, drug effectiveness screening, and allow prediction of patient response to chemotherapy and radiotherapy [64,65].

Genome-wide association studies (GWAS) have shown variations within genes involved in growth, proliferation and other genes involved in the development of the tumor microenvironment. The Cancer Genome Atlas (TCGA) has characterized genetic alterations across 9125 samples and 33 cancer types, consisting of case-based characterization of pathways such as RTK-Ras, PI3K, Wnt, Notch and TGF-b among others [66]. Barriers to cancer treatment often involve variable differences in cancer types and lack of generalizability between individuals, and OoC provides a promising mechanism to enable the study of cancer at the personalized level [65].

### 5.3. Acceleration of Drug Research and Testing Procedures

Organoids and OoCs have potential in predicting patient responsiveness to chemotherapy and radiotherapy aiding in both research efforts and precision medicine [67]. Using spheroid culture models in adjunct to microfluidics, generalizability to patient-specific tumor microenvironments may be achieved [68]. OoCs allow for the use of vascularized micro-tumors by co-culturing with additional cell types such as adipocytes, fibroblasts, and leukocytes, allowing for greater replicability of cell–cell communication and extracellular matrix conditions found in vivo [69,70,71]. In clinical practice, radiation and chemotherapeutic dosing may be titrated in patient-obtained cell cultures using OoCs to generate dose–response curves, optimize medication administration, and aid in treatment regimens based on side-effect profiles improving progression-free survival (PFS).

Pathogenic modeling may allow for assessing treatment options for various treatments in tissue cultures arising from lung, heart, brain, skin, liver and more. This allows for the evaluation of a multitude of disease processes not just limited to cancer but also progressions of pulmonary fibrosis, multiple sclerosis, ischemic heart disease, psoriasis, and non-alcoholic fatty liver disease to name a few.

Organ-on-a-chip technology also facilitates drug repurposing research. OoCs allow for high-throughput testing and the ability to screen many different drugs to identify which is more efficacious for repurposing [72]. An organ-on-a-chip experiment using an organ chip of human respiratory epithelium was able to screen existing antivirals and identify which were more efficacious at preventing infection by SARS-CoV-2 spike protein-expressing pseudoparticles. Identifying drugs that can be repurposed for pandemics and acute biothreat crises is particularly useful using this technology as testing can rapidly identify possible treatments and when waiting to fully develop a novel treatment is not feasible [72].

### 5.4. Precision Therapy and Drug Selection Advantages in Tumor and Cancer Research

Similarities in properties between microenvironments in OoCs and patient-specific solid tumors aid in generating precision therapy for individuals. Genetic aberrations as mentioned earlier serve as a barrier to creating generalized therapy when treating tumor subtypes with variable genetic aberrations. As mutations in PI3K, MAPK, BRAF associated with EGFR associated mutations are prevalent across a multitude of cancers, OoC may be used to assess patient-specific sensitivity to MEK inhibitors, BRAF inhibitors, and EGFR/RTK inhibitors [40]. Evaluating cellular crosstalk across numerous cell types in vitro is limited to strategies such as co-culture experiments with trans well inserts, and OoCs may address this issue by culturing multiple tissue types in synergy. In addition, replicating tumor vasculature within OoCs allows for testing of sensitivity of VEGF-inhibitors such as bevacizumab and ramucirumab. As cancer sensitivity to anti-tumor medication is variable, OoCs may be used to evaluate multi-drug resistance [73].

OoCs constructed with patient-derived induced pluripotent stem cells (iPSCs) will be particularly useful in the future of precision medicine as they contain cells with a patient’s specific genetic background. This allows for the study of patient-specific testing of drug efficacy or toxicity. There has however been limited success in generating iPSC cancer models due to challenges in both generating iPSCs from neoplastic cells and in differentiating iPSCs into the desired cell type [74].

Immunotherapy, while effective against some types of solid tumors, has greater efficacy against hematological malignancies. Current immunotherapeutic regimens include a combination of anti-CTLA-4, anti-PD-1/PD-L1, and CAR (Chimeric Antigen Receptors) T-cell recombinant therapy [66,75]. As OoCs lack integration with the immune system, modifying traditional OoCs to utilize an air-liquid interface may allow for the study of immunotherapy using peripheral lymphocytic co-culture [76].

### 5.5. Challenges in Implementation

While promising, patient-on-chip technologies still face some challenges to implementation. One such challenge is a variability in patient-derived cells due to cells being taken from different sources or reprogramming [77]. Protocols should standardize the procurement source for donor cells to limit potential for variability, as variability can lead to inconsistent responses. Additionally, with such an individualized technology, each batch of cell preparations may feature variations. Current OoC/ToC technologies are labor-intensive and require complex fabrication; automated approaches to OoC/ToC design will need to be developed for more scalability and before routine clinical use of these systems can be considered [19].

## 6. Conclusions

OoC/ToC technologies are transformative advancements in regenerative medicine and translational research that offer unprecedented opportunities to closely mimic human organ physiology. Beyond mere replication of physiologic conditions, these platforms enable personalized disease modeling and drug screening without reliance on animal models. Despite their promise, the development of OoC/ToC technologies remains a challenge. Successful design requires complex integration of various disciplines including materials science, cell biology, engineering, and medicine.

Engineered microfluidic systems can replicate aspects of human organ physiology and even the intricate interactions between human organs in the case of multi-organ-on-chip models. Compared to two-dimensional systems of modelling human physiology with cell culture, OoC/ToC devices operate on a three-dimensional scale more similar to true organs, all without requiring the use of live tissue or animals for research discoveries. These technologies offer improvements in automation, streamline data analysis processes, offer cost-effective options like paper microfluidics, and even enable single-cell analysis. The ability of ToC/OoC technologies to provide more individualized, patient-specific disease modeling also enhances their applicability for drug screening and disease modeling.

Currently, not all fields are equally suited to in vitro models, and the complexity of the immune system responses, behavior, hormonal responses, and development have yet to be effectively replicated by ToC models. Further research will be required if ToC/OoC is to model these complex systemic interactions within the human body. Cost is an additional barrier, and not all research facilities have the resources to initially create intricate ToC systems.

OoC microfluidic systems offer a promising alternative to animal testing for drug efficacy screening by simulating specific human organ functions, such as those of the liver, lung, heart, and bone. While the promise of OoC/ToC systems replacing animal testing is compelling, these systems will require an initial investment to further optimize and develop these systems before their widespread implementation. Additionally, implementation will require standardization of these models, as they have less robust data than historical animal models regarding expected outcomes and validated procedures. As ToC technologies develop, animal testing may be progressively replaced with in vitro systems that have the capacity to mimic human physiology as well as or better than animal models.

OoC/ToC systems are advancing medicine and providing new research options for personalized medicine, regenerative medicine, precise pathophysiology monitoring, drug safety studies, and eventual research cost savings. Further development to address their challenges could allow for widespread implementation of these systems and a significant shift in medical research methods.

## Data Availability

Not applicable.
